# Development, Electrochemical Characterization, and Statistical Optimization of a Low-Cost Membraneless Tubular Flow-By Alkaline Electrolyzer for Green Hydrogen Production

**DOI:** 10.3390/molecules31152688

**Published:** 2026-08-02

**Authors:** Bruno Augusto Cabral Roque, Hugo Morais Meira, Valdemir Alexandre dos Santos, Mohand Benachour, Leonie Asfora Sarubbo

**Affiliations:** 1 Department of Chemical Engineering, Federal University of Pernambuco (UFPE), Av. dos Economistas, s/n, Recife 50740-590, Brazil; bruno.roque@ufpe.br (B.A.C.R.); mohand.benachour@ufpe.br (M.B.); 2 Advanced Institute of Technology and Innovation (IATI), Rua Potira, 31, Recife 50751-310, Brazil; hugo.meira@iati.org.br (H.M.M.); valdemir.santos@unicap.br (V.A.d.S.); 3 School of Technology and Communication, Catholic University of Pernambuco (UNICAP), Rua do Príncipe, 526, Recife 50050-900, Brazil

**Keywords:** bubble removal, design of experiments, energy efficiency, gas–liquid separation, hydrodynamic optimization, polarization curves, response surface methodology

## Abstract

Hydrogen production by alkaline water electrolysis is a promising pathway for sustainable energy systems; however, conventional electrolyzers rely on membranes or diaphragms that increase cost, electrical resistance, and maintenance requirements. This study reports the development and experimental evaluation of a low-cost, membrane-less, flow-by tubular alkaline electrolyzer integrated with a dedicated gas–liquid separation and volumetric gas-measurement system. The electrolyzer was designed, constructed, experimentally validated, and electrochemically characterized using polarization curves. The effects of KOH concentration (0.50–2.00 mol L^−1^), electrolyte flow rate (1.00–4.00 L min^−1^), and applied voltage (2.20–3.20 V) on hydrogen production were investigated through a full factorial 3^3^ experimental design combined with analysis of variance, response surface methodology, and numerical optimization using the desirability function. The applied voltage was identified as the dominant operating variable, followed by KOH concentration and electrolyte flow rate, while significant interactions involving voltage were also observed. The quadratic regression model showed excellent predictive performance (R^2^ = 0.9614), and within the investigated operating domain, the highest hydrogen production rate was obtained at 2.00 mol L^−1^ KOH, 1.00 L min^−1^ electrolyte flow rate, and 3.20 V, resulting in an experimental hydrogen production rate of 0.4395 mL s^−1^. These findings demonstrate the potential of the proposed membrane-less flow-by electrolyzer as a simplified, low-cost, and experimentally validated platform for investigating and optimizing membrane-less alkaline water electrolysis under the evaluated operating conditions.

## 1. Introduction

Global energy demand continues to be predominantly met by fossil fuels, particularly natural gas, coal, and petroleum, sustaining a carbon-intensive model responsible for a significant share of anthropogenic greenhouse gas (GHG) emissions. This pattern of energy production and consumption directly contributes to the intensification of global warming and increases the risk of exceeding the critical 1.5 °C threshold, a scenario associated with worsening, potentially irreversible climate impacts. In this context, climate change has become one of the major global challenges of the twenty-first century, with systemic implications for environmental stability, economic development, food security, and public health [1,2,3].

The energy transition represents a structural transformation of energy systems, driven by the progressive replacement of fossil fuels with renewable energy sources and low-carbon technologies. This process involves not only technological innovation but also economic, institutional, and sociopolitical changes, requiring coordinated action among governments, industry, and society [4,5]. In this context, the intensification of global commitments to net-zero emissions has increased interest in sustainable energy solutions, positioning hydrogen (H_2_) as a strategic energy carrier for decarbonization, particularly in hard-to-abate sectors [6,7].

Hydrogen stands out for its high gravimetric energy density, versatility of applications, and absence of carbon emissions at the point of use. It can be produced through different pathways, stored, transported, and converted into final energy. However, its environmental performance depends directly on the production pathway, since hydrogen does not occur naturally in its elemental state. In this context, hydrogen production technologies are classified by colors according to their origin and associated emission intensity. Among the most relevant pathways, gray hydrogen, produced from fossil fuels, is characterized by high CO_2_ emissions; blue hydrogen incorporates carbon capture technologies; and green hydrogen, produced by water electrolysis using renewable electricity, represents the alternative with the lowest environmental impact [8,9,10].

Water electrolysis is one of the main pathways for green hydrogen production, based on the electrochemical decomposition of water into hydrogen (H_2_) and oxygen (O_2_) using electric current, particularly when integrated with renewable energy sources such as solar and wind power [11]. The main electrolysis technologies include alkaline water electrolysis (AWE), proton exchange membrane electrolysis (PEM), anion exchange membrane electrolysis (AEM), and solid oxide electrolysis cells (SOECs), which differ in their electrolytes, materials, and operating conditions [12,13]. Among these technologies, alkaline water electrolysis is the most mature and widely employed at the industrial scale, being characterized by high robustness, relatively low cost, and the use of metallic electrodes immersed in alkaline electrolytes, such as KOH or NaOH, separated by a porous diaphragm that enables ionic conduction while limiting gas crossover. These systems typically operate at moderate temperatures, with cell voltages ranging from 1.8 to 2.4 V and efficiencies between 62 and 82% [14,15].

Despite the widespread use of membranes and diaphragms in conventional electrolyzers, these components increase system costs, introduce additional resistance to ionic transport, and are prone to degradation during operation. Furthermore, separation components represent a significant fraction of the overall cost of electrolytic systems and require periodic maintenance and replacement throughout the equipment’s service life. In response to these limitations, membrane-less electrolyzers have been investigated as promising alternatives that simplify system design, reduce costs associated with separation components, and improve operational durability [16,17].

Among the different configurations proposed for membrane-less electrolyzers, flow-by systems have received particular attention. In these systems, the electrolyte flows through the electroactive region, promoting the transport of the products generated during electrolysis. This architecture belongs to the class of active separation systems, in which the electrolyte flow plays a fundamental role in the segregation and removal of the electrochemically generated products, representing a promising alternative for the development of simpler, more scalable, and potentially more cost-effective electrolyzers [18,19].

Despite its technological maturity, alkaline water electrolysis still presents limitations that affect its efficiency, including high overpotentials associated with the kinetics of the hydrogen evolution reaction, ohmic losses, and mass transport limitations. One of the most critical factors is the formation, growth, and adhesion of hydrogen and oxygen bubbles on the electrode surfaces, which reduce the electrochemically active area, hinder ionic transport, and increase resistance by blocking conductive pathways within the electrolyte. The bubble detachment dynamics, influenced by the electrolyte properties, current density, and hydrodynamic conditions, are a key determinant of system performance. In this context, strategies based on improved reactor design and control of hydrodynamic conditions have been investigated to promote bubble removal from electrode surfaces, reduce mass-transport limitations, and enhance the overall performance of electrolysis systems [20,21]. 

In this context, the development of low-cost alkaline electrolyzers based on simplified architectures represents a promising strategy to enhance the feasibility of sustainable hydrogen production. Although recent advances in membrane-less systems have demonstrated potential for cost reduction, simplified construction, and decreased losses associated with separation components, challenges related to hydrodynamic control, mass transport, removal of the generated gases, and operational efficiency still limit their technological consolidation [22]. 

Despite significant advances in membrane-less water electrolysis, current research has predominantly focused on membrane-less flow-by electrolyzers, emphasizing hydrodynamic control, gas crossover mitigation, bubble transport, reactor design, numerical modeling, and electrochemical performance under representative operating conditions. In membrane-less flow-by electrolyzers, the electrolyte simultaneously provides ionic conduction and acts as a dynamic separator. Controlled electrolyte flow promotes convective transport, facilitates bubble removal, and minimizes hydrogen and oxygen crossover. Although these studies have substantially advanced understanding of hydrodynamic behavior and flow-mediated gas separation, they have primarily addressed specific engineering challenges rather than systematic investigations that integrate reactor development, electrochemical characterization, statistical optimization, and comprehensive energy-performance evaluation [23,24]. Furthermore, studies on tubular electrochemical flow cells have demonstrated that reactor geometry and multiphase hydrodynamics strongly influence mass transport and electrochemical performance; however, these concepts have been explored mainly in other electrochemical systems, highlighting the need for integrated experimental validation of tubular membrane-less flow-by alkaline electrolyzers [25,26].

Design of Experiments (DoE) has become an increasingly important statistical framework for the systematic development and optimization of electrolysis systems. By enabling the simultaneous evaluation of multiple operating variables with fewer experimental runs, DoE provides a structured approach for identifying the individual and interactive effects of process parameters while improving experimental efficiency and resource utilization. When combined with Response Surface Methodology (RSM), DoE enables the development of predictive models, the quantification of factor interactions, and the identification of optimal operating conditions through statistically robust optimization. Consequently, the integration of DoE and RSM has become an effective strategy for the systematic development, evaluation, and optimization of electrochemical systems [27,28,29]. 

In parallel, additive manufacturing has emerged as an attractive fabrication strategy for electrochemical devices because it enables rapid prototyping, high geometric flexibility, simplified design modifications, reduced manufacturing costs, and the fabrication of complex flow-field architectures that can be experimentally validated before scale-up [30]. Recent studies have successfully integrated three-dimensional (3D) printing into electrochemical reactor development, enabling the production of multifunctional structural components and customized flow channels while accelerating design iteration, reactor development, and experimental validation [31]. Rather than constituting the primary scientific contribution, additive manufacturing serves as an enabling technology that facilitates modular reactor development, rapid prototyping, and the establishment of adaptable, low-cost experimental platforms for systematic investigations under different operating conditions.

To address these challenges, the present study reports the development and experimental validation of a low-cost, tubular, membrane-less flow-by alkaline electrolyzer that incorporates additively manufactured structural components and is integrated with a dedicated gas–liquid separation and volumetric gas measurement system, enabling independent quantification of hydrogen and oxygen production. The reactor was designed, fabricated, experimentally validated, and subsequently electrochemically characterized using polarization curves. This integrated platform enabled a comprehensive experimental framework encompassing reactor development, electrochemical characterization, gas management, and systematic optimization of operating conditions. The effects of KOH concentration, electrolyte flow rate, and applied voltage on hydrogen production were investigated using a full 3^3^ factorial design, combined with analysis of variance, response surface methodology, and numerical optimization. Finally, electrochemical and energy-performance indicators were determined under the optimum operating conditions. By integrating modular reactor design, additive manufacturing, dedicated gas management, electrochemical characterization, statistical optimization, and energetic assessment within a single experimentally validated platform, this work provides a comprehensive experimental framework for the development and evaluation of simplified, modular, and cost-effective membrane-less alkaline electrolysis systems for sustainable hydrogen production.

## 2. Results

### 2.1. Polarization Curves

**Figure 1:**
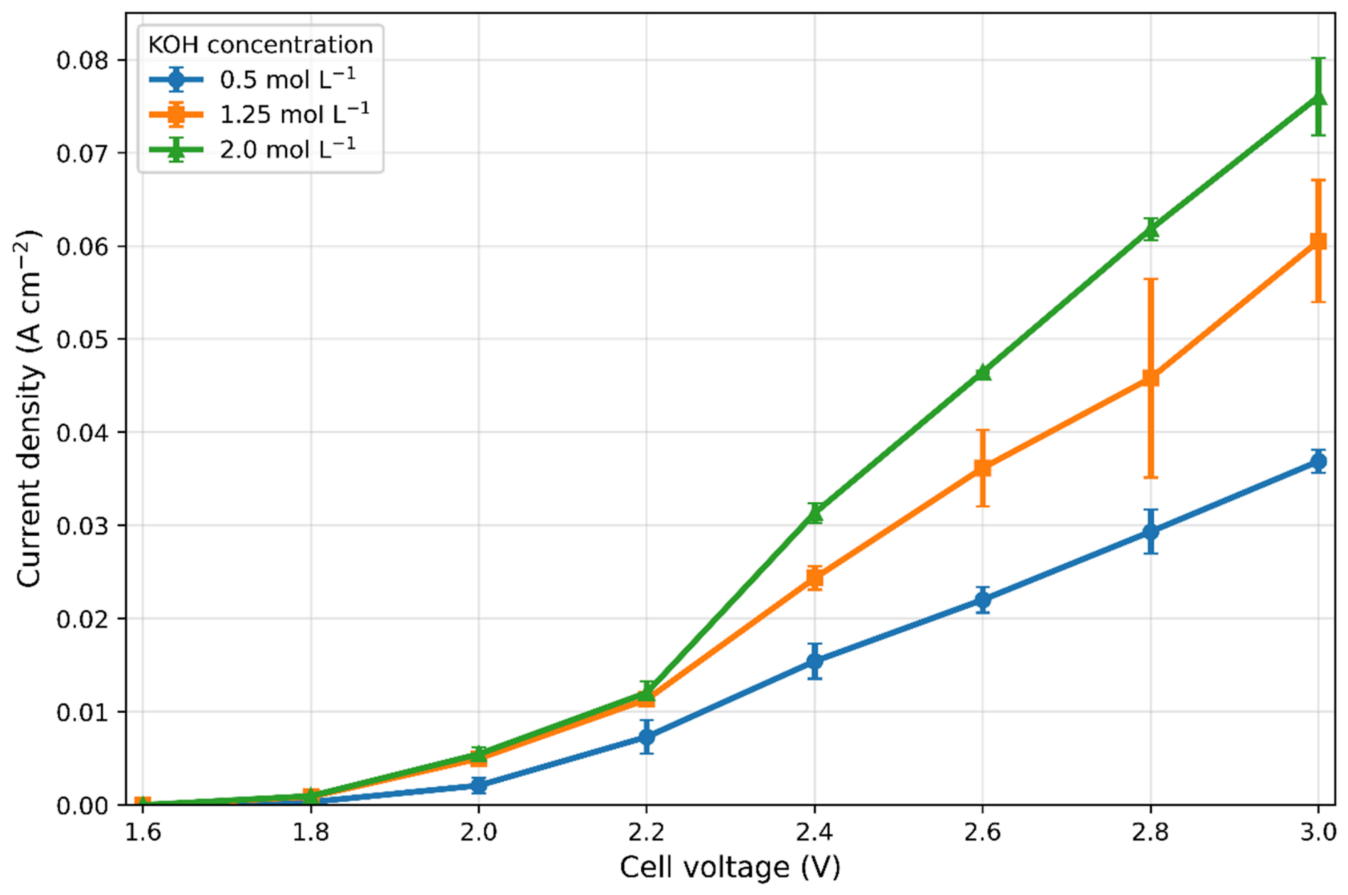
Polarization curves of the membrane-free flow-by alkaline electrolyzer operating at an electrolyte flow rate of 2.50 L min^−1^ and KOH concentrations of 0.50, 1.25, and 2.00 mol L^−1^.

Figure 1 presents the polarization curves obtained for the membrane-free flow-by alkaline electrolyzer operating at a fixed electrolyte flow rate of 2.5 L min^−1^, using KOH concentrations of 0.50, 1.25, and 2.00 mol L^−1^. The curves were constructed from the average current density across three independent measurements at each operating voltage, with error bars representing the corresponding standard deviations.

The polarization curves exhibited the characteristic electrochemical behavior of alkaline water electrolysis, with current density increasing continuously as the applied cell voltage increased from 1.6 to 3.0 V. Regardless of electrolyte concentration, negligible current densities were observed below approximately 1.8 V, indicating that the applied potential was insufficient to overcome the activation overpotentials associated with the hydrogen evolution reaction (HER) and oxygen evolution reaction (OER). Above this threshold, current density increased progressively, reflecting enhanced electrode kinetics and electrolysis rates.

Electrolyte concentration markedly influenced cell performance over the voltage range investigated. Above 2.0 V, the 2.00 mol L^−1^ KOH solution consistently exhibited the highest current densities, followed by 1.25 mol L^−1^, whereas 0.50 mol L^−1^ produced the lowest values. At 3.0 V, the average current densities reached approximately 0.076, 0.060, and 0.037 A cm^−2^ for 2.00, 1.25, and 0.50 mol L^−1^, respectively, demonstrating that increasing the electrolyte concentration from 0.50 to 2.00 mol L^−1^ nearly doubled the current density under identical operating conditions.

The superior performance at higher KOH concentrations is attributed to the increased ionic conductivity of the electrolyte, which reduces ohmic resistance and minimizes voltage losses during ionic transport. As a result, a greater fraction of the applied potential is available to drive the electrochemical reactions, improving overall cell performance. This effect became increasingly evident above approximately 2.4 V, where the polarization curves diverged substantially. In contrast, below 2.2 V, the curves remained relatively close, suggesting that activation polarization was the predominant limitation in this voltage region.

The nearly linear increase in current density observed for the 2.00 mol L^−1^ electrolyte between 2.4 and 3.0 V indicates that the electrolyzer did not reach a mass-transfer-limited regime within the investigated operating window. The fixed electrolyte flow rate likely promoted efficient electrolyte renewal and bubble removal, minimizing concentration gradients and maintaining favorable reaction conditions. Conversely, the lower slope observed with 0.50 mol L^−1^ KOH reflects the greater internal resistance of the dilute electrolyte, leading to higher ohmic losses and reduced electrochemical efficiency.

The relatively small error bars observed across most operating conditions demonstrate good experimental repeatability. Although slightly greater variability was observed for 1.25 mol L^−1^ at voltages above 2.6 V, likely due to the dynamic nature of gas bubble formation and detachment, these variations remained small compared with the differences among electrolyte concentrations.

Overall, the polarization curves show that increasing KOH concentration significantly enhances the electrochemical performance of the membrane-free flow-by electrolyzer by lowering internal resistance and enabling higher current densities. Although polarization curves provide a comprehensive electrochemical assessment of cell performance, additional quantitative information on the electrolyzer’s overall electrical behavior can be obtained from the current–voltage relationship. Therefore, an apparent electrical characterization based on linear regression of the polarization data was performed to determine complementary electrical descriptors of the developed system.

### 2.2. Apparent Electrical Performance

To further quantify the electrical behavior observed in the polarization curves, the approximately linear region of the current–voltage relationship was analyzed using linear regression. This complementary analysis provides quantitative descriptors of the electrolyzer’s overall electrical performance, enabling direct comparison of the apparent electrical conductance, apparent electrical resistance, and apparent geometric conductivity across different electrolyte concentrations.

**Figure 2:**
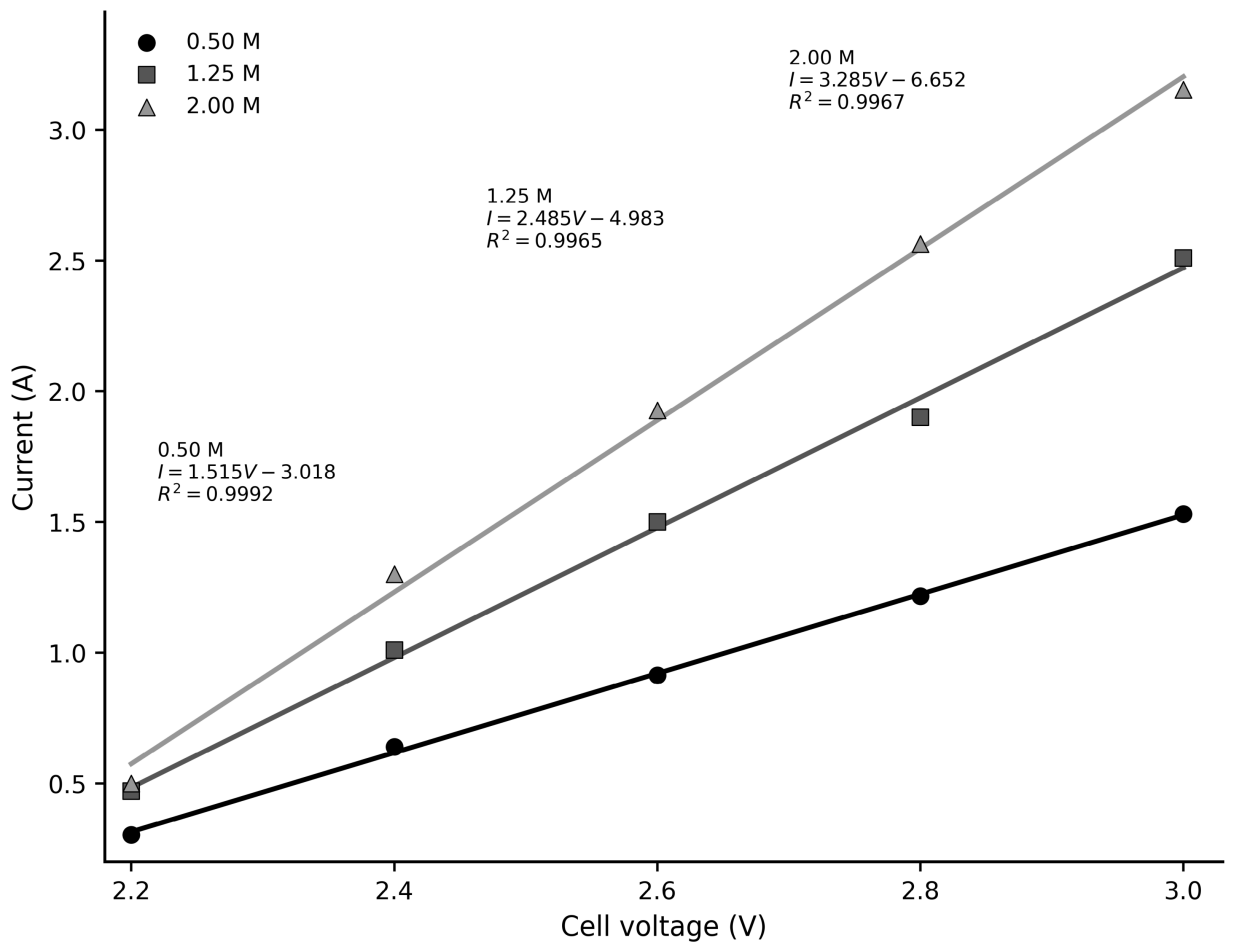
Linear regression of the current–voltage relationship in the approximately linear region of the polarization curves for different KOH concentrations. The slope of each regression line was used to estimate the apparent electrical conductance of the membrane-free flow-by alkaline electrolyzer.

The linear regressions for each electrolyte concentration are shown in Figure 2. The current–voltage relationship was highly linear across the selected voltage range for all investigated electrolyte concentrations, with coefficients of determination (R^2^) greater than 0.996, confirming that the selected operating region provides a robust basis for estimating the apparent electrical conductance of the electrolyzer.

As shown in Figure 2, the current–voltage relationship was highly linear across the selected operating region for all investigated electrolyte concentrations. The systematic increase in the slope of the regression lines with increasing KOH concentration indicates a progressive enhancement in the apparent electrical conductance of the electrolyzer, reflecting improved ionic transport within the electrolyte. The corresponding apparent electrical parameters derived from these regressions are summarized in Table 1. 

As shown in Table 1, the apparent electrical conductance increased from 1.515 to 3.285 S as the KOH concentration rose from 0.50 to 2.00 mol L^−1^, while the corresponding apparent electrical resistance decreased from 0.660 to 0.304 Ω. Likewise, the apparent geometric conductivity increased steadily from 0.0653 to 0.1416 S cm^−1^, indicating a progressive improvement in overall electrical conduction within the electrolyzer.

These quantitative results are in excellent agreement with the electrochemical behavior observed in the polarization curves. Increasing the KOH concentration increases the number of ionic charge carriers in the electrolyte, thereby facilitating ionic transport between the electrodes and reducing electrical losses within the cell. Consequently, a larger fraction of the applied potential drives the hydrogen and oxygen evolution reactions, yielding higher current densities under identical operating conditions. This behavior aligns with the progressive separation of the polarization curves at higher applied voltages and confirms the beneficial effect of electrolyte concentration on the electrical response of the membrane-free flow-by alkaline electrolyzer.

The apparent electrical characterization therefore complements the interpretation of the polarization curves by providing quantitative electrical descriptors of the overall cell response. These results establish the electrical basis for interpreting how electrolyte concentration influences electrolyzer performance and provide additional support for the subsequent statistical evaluation of the combined effects of electrolyte concentration, electrolyte flow rate, and applied voltage on hydrogen production, using the full factorial experimental design.

### 2.3. Experimental Design and Statistical Analysis

The experimental responses obtained from the 3^3^ full factorial design with six additional replicated center points exhibited a wide variation in hydrogen production rate, ranging from 0.0118 to 0.4395 mL s^−1^ across the investigated operating conditions. The six additional center-point replicates showed excellent repeatability, with hydrogen production rates varying only between 0.1134 and 0.1139 mL s^−1^, confirming the high reproducibility of the experimental procedure and indicating that the observed response variability was primarily attributable to changes in operating variables rather than to experimental uncertainty.

**Figure 3:**
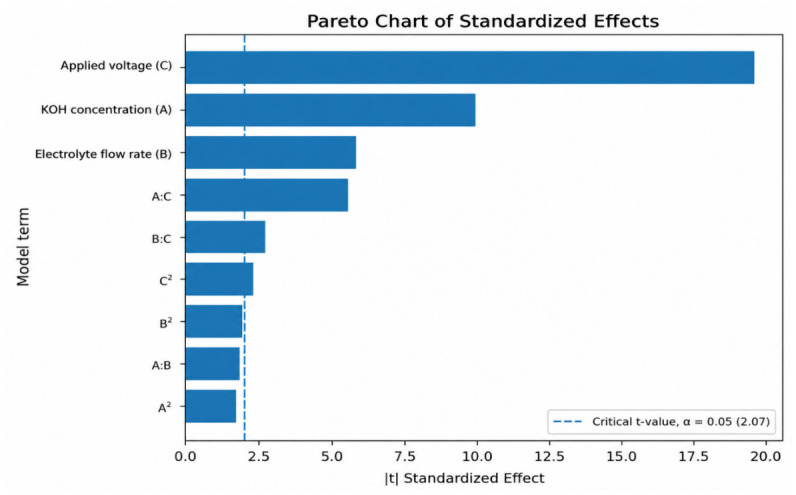
Standardized Pareto chart of the effects estimated from the quadratic response surface model for hydrogen production rate. The dashed vertical line represents the critical Student’s *t*-value (*t* = 2.07) at the 95% confidence level (α = 0.05). Model terms extending beyond this threshold were considered statistically significant.

An initial assessment of the influence of the investigated factors was performed using the standardized Pareto chart (Figure 3), which ranks the linear, interaction, and quadratic effects according to their statistical significance and relative magnitude. The vertical reference line represents the critical Student’s *t*-value (*t* = 2.07, α = 0.05), and all effects extending beyond this limit were considered statistically significant. 

As shown in the Pareto chart, the applied voltage (C) exhibited by far the largest standardized effect, demonstrating that the electrical potential is the dominant variable controlling hydrogen production within the investigated operating domain. This result is consistent with the electrochemical nature of alkaline water electrolysis, in which increasing the applied voltage enhances the current density and thereby accelerates the hydrogen evolution reaction.

The KOH concentration (A) was identified as the second most influential factor, highlighting the importance of electrolyte conductivity in reducing ohmic resistance and improving charge transport. The electrolyte flow rate (B) also exerted a significant effect on the response, indicating that hydrodynamic conditions contribute to efficient electrolyte renewal and gas bubble removal from the electrode surface, thereby improving the electrolysis performance.

Among the interaction effects, the A × C (KOH concentration × voltage) interaction exhibited the largest standardized effect, indicating a strong synergistic relationship between electrolyte conductivity and the applied electrical potential. This behavior indicates that the beneficial effect of increasing the applied voltage becomes more pronounced under highly conductive electrolyte conditions. The B × C (flow rate × voltage) interaction was also statistically significant, whereas the A × B interaction remained below the significance threshold, suggesting that KOH concentration and electrolyte flow rate act predominantly independently within the investigated operating range.

Regarding the quadratic terms, only the voltage quadratic effect (C^2^) exceeded the statistical significance threshold, providing the first indication that hydrogen production exhibits nonlinear behavior with respect to the applied voltage. In contrast, the quadratic effects associated with KOH concentration and electrolyte flow rate were comparatively small and did not reach statistical significance.

Overall, the Pareto analysis demonstrates that hydrogen production in the membrane-free flow-by electrolyzer is governed primarily by the applied voltage, followed by electrolyte concentration and electrolyte flow rate, while significant synergistic interactions between voltage and the remaining operating variables further influence the process performance. These preliminary findings justify the subsequent application of analysis of variance (ANOVA) to quantitatively evaluate the contribution of each model term and validate the fitted quadratic response surface model.

### 2.4. Analysis of Variance (ANOVA)

The statistical significance of the quadratic response surface model was evaluated by analysis of variance (ANOVA), and the corresponding results are summarized in Table 2. ANOVA was used to determine the contribution of the linear, interaction, and quadratic terms of KOH concentration (A), electrolyte flow rate (B), and applied voltage (C) to the hydrogen production rate. Statistical significance was defined at the 95% confidence level (*p* < 0.05).

The quadratic regression model was highly significant (*F* = 63.72, *p* < 0.0001), indicating that the selected polynomial adequately described the relationship between the investigated operating variables and the hydrogen production rate. The relatively small residual variation relative to the model sum of squares indicates that most of the experimental variability was explained by the fitted regression model.

Among the linear effects, the applied voltage (C) was identified as the dominant factor governing hydrogen production, exhibiting the highest *F*-value (*F* = 384.53, *p* < 0.0001). This finding confirms that increasing the applied electrical potential substantially enhances the electrochemical kinetics of the hydrogen evolution reaction by increasing the current density available for water electrolysis. The KOH concentration (A) also exerted a highly significant positive effect (*F* = 99.36, *p* < 0.0001), demonstrating that improved electrolyte conductivity reduces ohmic resistance and favors hydrogen generation. Likewise, the electrolyte flow rate (B) significantly affected the response (*F* = 34.65, *p* < 0.0001), highlighting the importance of electrolyte circulation for enhancing mass transport and the efficient removal of gas bubbles from the electrode surface.

The interaction analysis revealed that the A × C interaction was highly significant (*F* = 31.26, *p* < 0.0001), indicating a strong synergistic relationship between electrolyte conductivity and applied voltage. This result demonstrates that the beneficial effect of increasing the applied voltage becomes considerably more pronounced at higher KOH concentrations. The B × C interaction was also statistically significant (*F* = 7.57, *p* = 0.0114), suggesting that the electrochemical response depends on the hydrodynamic operating conditions. In contrast, the A × B interaction was not significant (*p* = 0.0672), indicating that KOH concentration and electrolyte flow rate affect hydrogen production predominantly through independent mechanisms within the investigated operating region.

Regarding the quadratic effects, only the voltage quadratic term (C^2^) remained statistically significant (*F* = 5.45, *p* = 0.0286), confirming that the response exhibits nonlinear behavior with respect to the applied voltage. Conversely, the quadratic terms associated with KOH concentration (A^2^) and electrolyte flow rate (B^2^) were not statistically significant, indicating that their effects remain approximately linear over the experimentally investigated domain.

Overall, the ANOVA demonstrates that hydrogen production in the proposed membrane-free flow-by alkaline electrolyzer is primarily controlled by the applied voltage, followed by KOH concentration and electrolyte flow rate. Furthermore, the significant interactions involving applied voltage emphasize that simultaneous optimization of the electrical and hydrodynamic operating conditions is essential for maximizing hydrogen production. The quadratic regression model explained more than 96% of the experimental variability and exhibited excellent statistical significance (*p* < 0.0001), providing a robust basis for developing the response surface model for process interpretation and numerical optimization.

### 2.5. Development and Validation of the Response Surface Model

Based on the statistically significant ANOVA results, a second-order polynomial regression model was developed to describe the combined effects of KOH concentration (A), electrolyte flow rate (B), and applied voltage (C) on the hydrogen production rate. The resulting quadratic equation, expressed in terms of coded variables, is presented in Equation (1):

Y = 0.13374574 + 0.06261700A − 0.03697875B + 0.12318094C − 0.01478136AB + 0.04301521AC − 0.02116472BC − 0.01773576A^2^ + 0.01974697B^2^ + 0.02309188C^2^ (1)

where A, B, and C represent the coded values of KOH concentration, electrolyte flow rate, and applied voltage, respectively.

The developed model exhibited excellent predictive performance, with a coefficient of determination (R^2^) = 0.9614, an adjusted coefficient of determination (R^2^adj) = 0.9464, and a predicted coefficient of determination (R^2^pred) = 0.9072. The relatively small difference between the adjusted and predicted coefficients indicates that the model has good predictive capability, with no evidence of substantial overfitting. Furthermore, the low prediction error (RMSE = 2.67 × 10^−2^ mL s^−1^) confirms the high accuracy of the fitted regression model within the investigated experimental domain.

**Figure 4:**
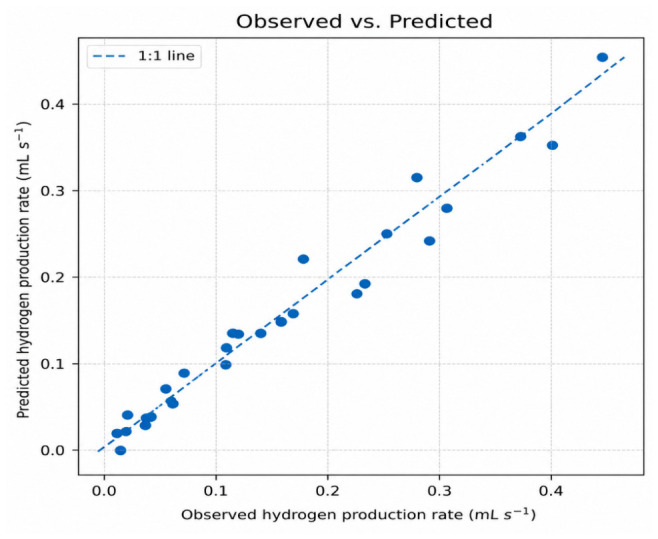
Observed versus Predicted Hydrogen Production Rate.

The predictive performance of the quadratic model is further demonstrated in Figure 4, which compares the experimental and model-predicted hydrogen production rates. The data points are closely distributed around the 1:1 reference line, indicating excellent agreement between measured and predicted values throughout the investigated response range. No systematic deviation or pronounced bias is observed, demonstrating that the model accurately reproduces the experimental behavior over both low and high hydrogen production rates. The slight dispersion observed for a few experimental points remains small relative to the overall response range and is consistent with the residual experimental variability, confirming the robustness of the fitted model. 

**Figure 5:**
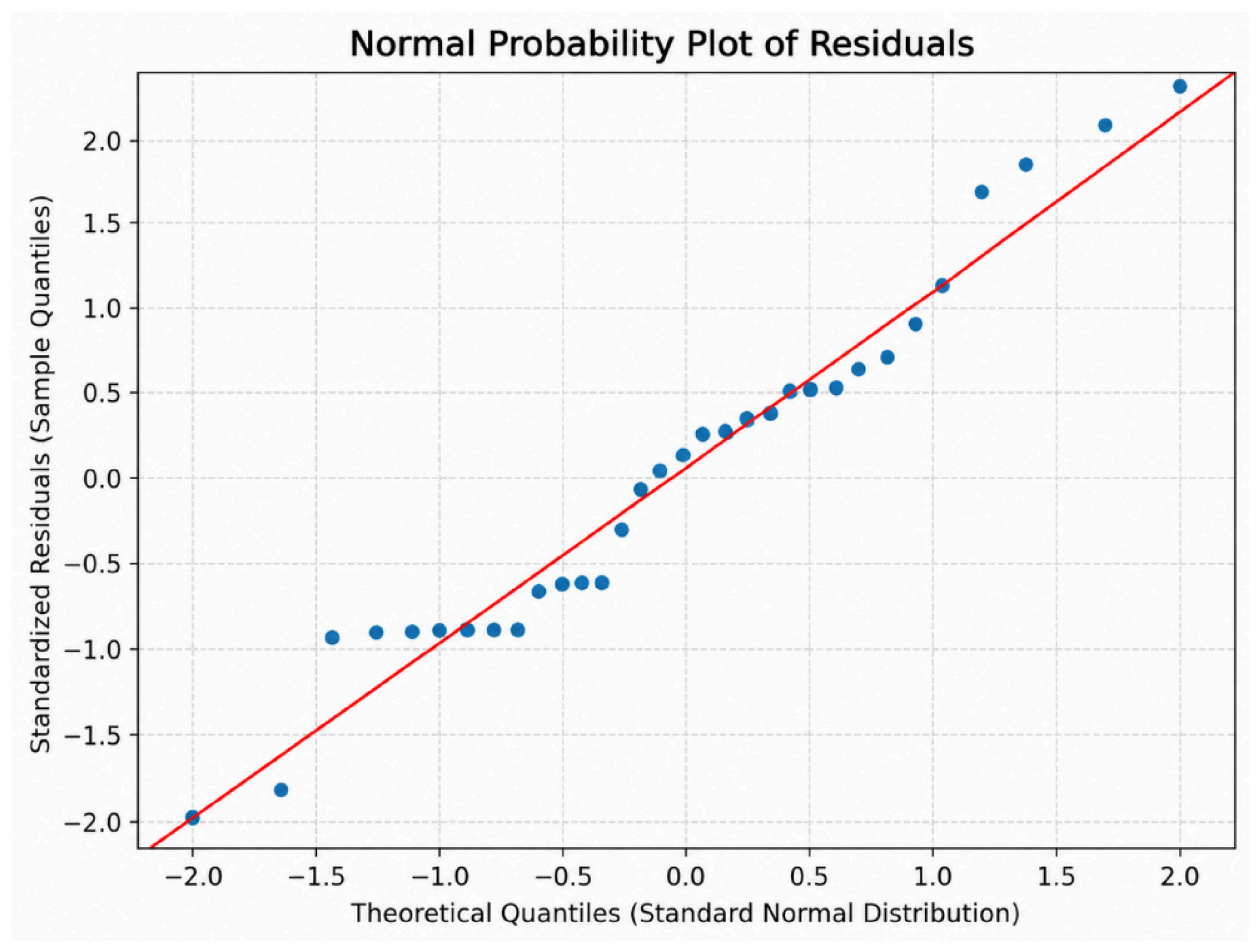
Normal Probability Plot of Residuals. The red line represents the theoretical normal distribution against which the standardized residuals are compared.

To verify the statistical assumptions underlying the regression analysis, the residuals were further evaluated using the normal probability plot shown in Figure 5. Most residuals closely follow the theoretical normal distribution, indicating that they are approximately normally distributed and that no severe violations of the normality assumption are present. Although slight deviations are observed at the extreme quantiles, these departures are limited to a few observations located at the tails of the distribution and are not sufficient to compromise the overall adequacy of the regression model. Therefore, the residual analysis supports the validity of the statistical inference and reinforces the reliability of the developed response surface model.

The curvature test was highly significant (*F* = 93.76, *p* < 0.001), confirming that the hydrogen production response exhibits pronounced nonlinear behavior over the experimental region investigated. This result demonstrates that a first-order approximation would be insufficient to adequately describe the process and justifies adopting a second-order polynomial model for response surface analysis.

Finally, numerical optimization based on the desirability function predicted the maximum hydrogen production rate at a KOH concentration of 2.00 mol L^−1^, an electrolyte flow rate of 1.00 L min^−1^, and an applied voltage of 3.20 V, corresponding to 0.4606 mL s^−1^. Because the optimum was located at the upper boundary of the investigated design space for both KOH concentration and applied voltage, these operating conditions represent the best performance attainable within the experimental region explored in this study. The optimization results are consistent with the response surface analysis and confirm that maximizing electrolyte conductivity while operating at the highest investigated voltage and the lowest electrolyte flow rate provides the most favorable conditions for hydrogen generation. Further improvements may be achieved by expanding the ranges of the investigated factors in future studies.

### 2.6. Response Surface Analysis

**Figure 6:**
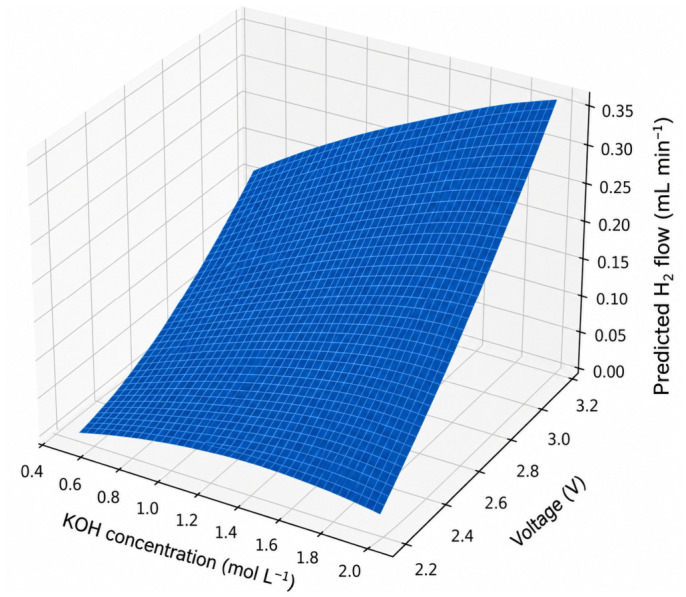
Response surface of hydrogen production rate as a function of KOH concentration and applied voltage.

The three-dimensional response surfaces were used to evaluate the combined effects of KOH concentration, electrolyte flow rate, and applied voltage on the hydrogen production rate. These plots provide a graphical interpretation of the fitted quadratic model and allow the identification of the operating regions associated with higher hydrogen production. The combined effect of KOH concentration and applied voltage on hydrogen production is shown in Figure 6.

**Figure 7:**
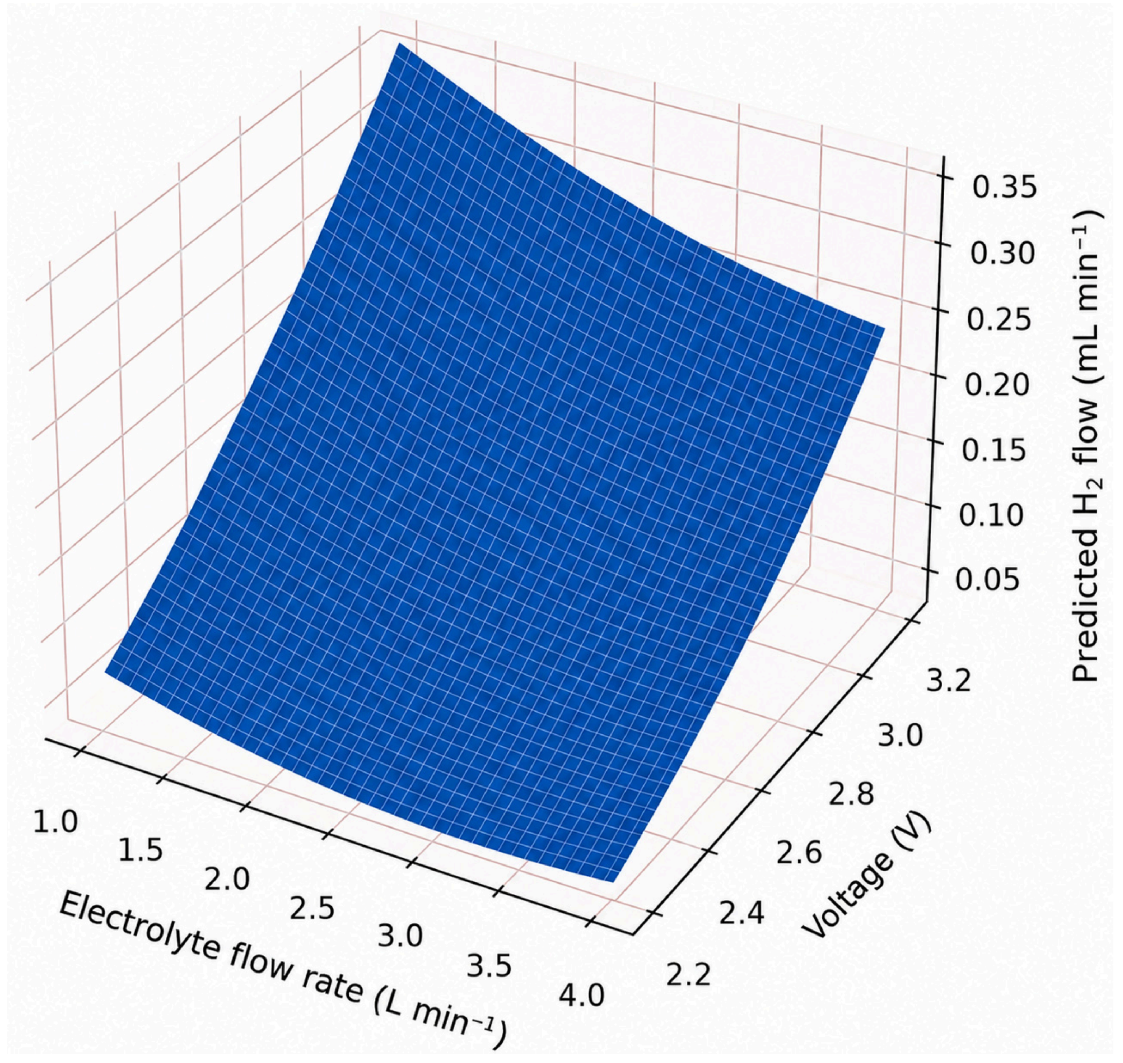
Response surface of hydrogen production rate as a function of electrolyte flow rate and applied voltage.

As shown in Figure 6, both KOH concentration and applied voltage positively influenced the hydrogen production rate. However, the effect of applied voltage was considerably more pronounced, as evidenced by the steeper increase in the response with increasing voltage. Hydrogen production increased continuously as the applied voltage was raised from 2.20 to 3.20 V, and further increasing the KOH concentration from 0.50 to 2.00 mol L^−1^ further enhanced the response. The highest predicted hydrogen production rates were obtained at the simultaneous combination of high KOH concentration and high applied voltage. This behavior is consistent with alkaline water electrolysis, since increasing the KOH concentration improves the electrolyte’s ionic conductivity and reduces ohmic losses, whereas increasing the applied voltage provides a greater driving force for the hydrogen evolution reaction. The curvature observed along the voltage axis is also consistent with the significant quadratic voltage term identified in the ANOVA. The combined effect of electrolyte flow rate and applied voltage is presented in Figure 7.

**Figure 8:**
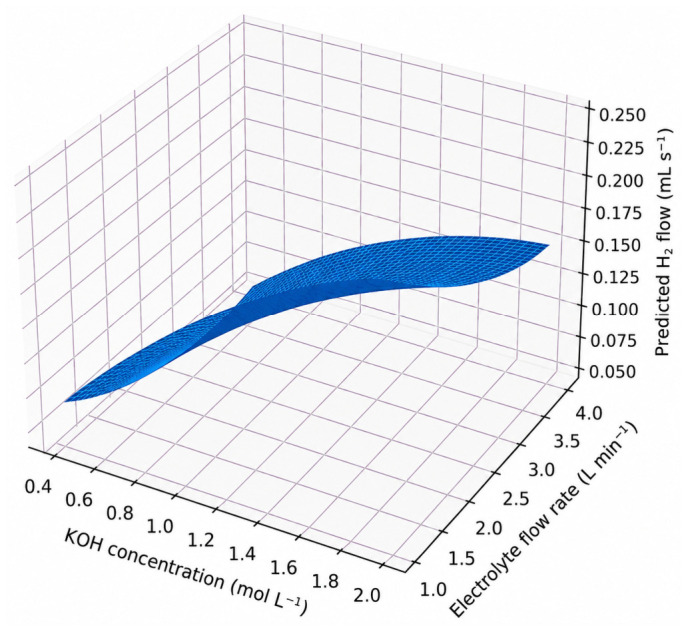
Response surface of hydrogen production rate as a function of KOH concentration and electrolyte flow rate.

As observed in Figure 7, the hydrogen production rate increased markedly with applied voltage but decreased with increasing electrolyte flow rate. Therefore, the highest predicted values were obtained under conditions of low electrolyte flow rate and high applied voltage. This trend suggests that, within the investigated flow range, increasing the electrolyte recirculation rate may reduce the electrolyte residence time in the reactor, thereby limiting effective electrochemical contact between the electrolyte and the electrode surface. Although higher flow rates can favor bubble removal and electrolyte renewal, in the present operating range, this benefit appears to be outweighed by the reduction in residence time. This result agrees with the negative effect of electrolyte flow rate and the significant B × C interaction observed in the ANOVA. The combined effect of KOH concentration and electrolyte flow rate on hydrogen production is shown in Figure 8.

Figure 8 shows that KOH concentration and electrolyte flow rate exerted opposite effects on hydrogen production. Increasing KOH concentration enhanced the response, whereas increasing electrolyte flow rate gradually reduced it. Compared with the previous surfaces, this plot exhibits only mild curvature, indicating a weaker interaction between these two factors. This observation is consistent with the non-significant A × B interaction identified by the ANOVA, suggesting that KOH concentration and electrolyte flow rate influenced hydrogen production predominantly through independent mechanisms within the experimental domain studied.

Overall, the response surface analysis confirms that hydrogen production in the membrane-free flow-by alkaline electrolyzer is primarily governed by applied voltage, followed by KOH concentration, while the electrolyte flow rate exerts a comparatively smaller, predominantly negative effect within the investigated range. The surfaces consistently indicate that the most favorable operating region corresponds to high KOH concentration, high applied voltage, and low electrolyte flow rate. These trends support the subsequent numerical optimization based on the desirability function.

### 2.7. Numerical Optimization Using the Desirability Function

**Figure 9:**
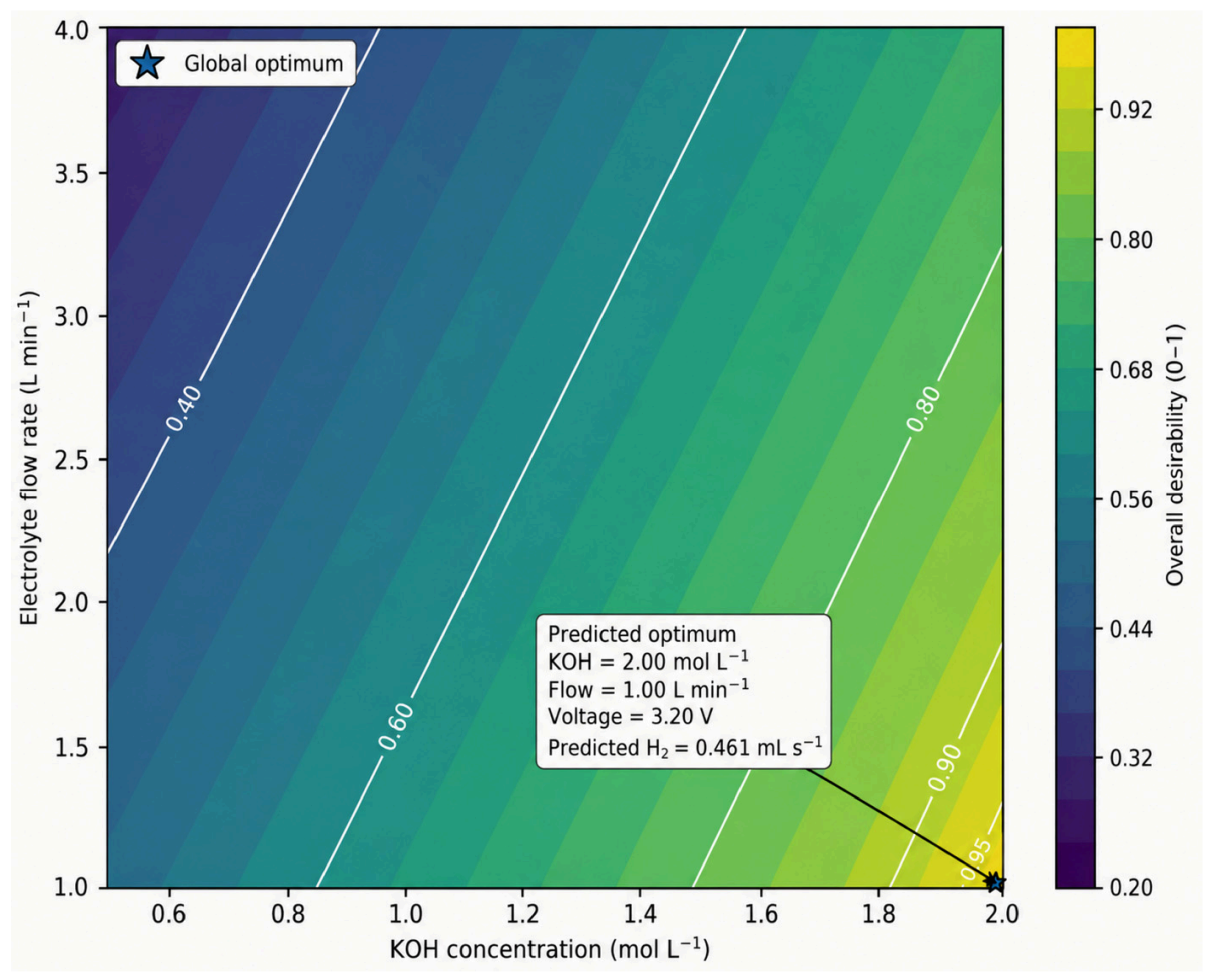
Overall desirability function for maximizing the hydrogen production rate as a function of KOH concentration and electrolyte flow rate, with the applied voltage fixed at 3.20 V. The star indicates the best predicted condition within the investigated experimental domain.

Following the response surface analysis, numerical optimization was performed using the desirability function approach to identify the operating conditions that maximize the hydrogen production rate within the investigated experimental domain. The optimization simultaneously considered the effects of KOH concentration, electrolyte flow rate, and applied voltage while maximizing the response predicted by the quadratic regression model. The optimization results are presented in Figure 9, which illustrates the overall desirability distribution as a function of KOH concentration and electrolyte flow rate, with the applied voltage fixed at 3.20 V.

As shown in Figure 9, the desirability progressively increased with increasing KOH concentration and decreasing electrolyte flow rate. The highest desirability values were concentrated in the lower-right region of the response map, corresponding to the simultaneous combination of high electrolyte concentration and low electrolyte flow rate. In contrast, low KOH concentrations combined with high electrolyte flow rates resulted in substantially lower desirability values, indicating operating conditions less favorable for maximizing the measured hydrogen production rate.

The numerical optimization identified the best predicted condition within the investigated domain at a KOH concentration of 2.00 mol L^−1^, an electrolyte flow rate of 1.00 L min^−1^, and an applied voltage of 3.20 V. Under these conditions, the quadratic model predicted a hydrogen production rate of 0.461 mL s^−1^. The experimentally measured value was 0.4395 mL s^−1^, corresponding to a relative deviation of approximately 4.7% from the model prediction. This agreement indicates that the model satisfactorily estimated the response at the selected validation condition. However, because the identified condition was located at the boundaries of the experimental domain for all three factors, it should be interpreted as the best condition identified within the investigated range rather than as an unrestricted global optimum.

To complement the experimental validation of the selected operating condition, the corresponding electrochemical and energy-performance indicators were computed from the measured voltage, current, and hydrogen-production data. The results are summarized in Table 3.

The selected operating condition yielded the highest experimentally measured hydrogen production rate across the investigated domain, with a current density of 0.0943 A cm^−2^ and a Faradaic efficiency of 88.7%. The cell-specific electrical energy consumption, calculated from the electrical power supplied to the electrolyzer and the hydrogen production rate normalized to standard conditions, was 8.63 kWh Nm^−3^ H_2_. This value reflects only the electrical energy supplied to the electrochemical cell and excludes auxiliary consumption by the pump, sensors, valves, microcontroller, and other balance-of-plant components.

The HHV-based energy efficiency was calculated to be 41.0%. This result is quantitatively consistent with the applied cell voltage and the experimentally determined Faradaic efficiency. Under ambient conditions, with a thermoneutral voltage of approximately 1.48 V, the theoretical voltage efficiency can be estimated using Equation (2):

(2) $$\eta_{voltage,HHV\;}=\frac{V_{th}}{V_{cell}}=\frac{1.48}{3.20}=0.4625$$

This corresponds to a theoretical voltage efficiency of approximately 46.3%. When the experimentally determined Faradaic efficiency is incorporated, the overall HHV-based energy efficiency can be independently estimated using Equation (3):

(3) $$\eta_{HHV\;}=\eta_{F\;}\frac{V_{th}}{V_{cell}}=0.887\times \frac{1.48}{3.20}\approx 0.410$$

Therefore, the calculated efficiency of approximately 41.0% is internally consistent with the electrochemical and gas-production measurements. Nevertheless, this value remains below the efficiency typically achieved by industrial alkaline electrolyzers and should not be interpreted as evidence of industrial-level energetic competitiveness.

The relatively low energy efficiency is primarily due to the high cell voltage required to achieve the maximum hydrogen production rate. The applied voltage includes the reversible potential of water electrolysis, activation overpotentials associated with the hydrogen and oxygen evolution reactions, ohmic losses across the electrolyte and electrical contacts, and additional resistance caused by gas-bubble coverage. These losses were exacerbated by the use of uncoated AISI 304 cylindrical rod electrodes, operation at room temperature, the limited geometric electrode area, and the non-optimized ionic distance between the electrodes.

The effects of the operating variables on hydrogen production reflect the combined influence of electrochemical conductivity and two-phase hydrodynamics. Increasing the KOH concentration improved ionic conductivity and reduced electrolyte resistance, thereby increasing the current and hydrogen production rate. Increasing the applied voltage provided a greater electrochemical driving force, although it also increased energy demand and reduced voltage efficiency. The lower hydrogen production measured at higher electrolyte circulation rates is attributed to changes in two-phase flow and gas-recovery behavior, including shorter gas–liquid disengagement times and possible microbubble entrainment, rather than solely to a reduction in electrolyte residence time.

Because the best operating condition was found at the highest investigated KOH concentration and applied voltage and the lowest electrolyte flow rate, the optimization did not identify an internal stationary optimum within the investigated experimental domain. Instead, it identified the most favorable operating condition among the evaluated factor combinations. Although extending the investigated operating ranges could potentially increase the hydrogen production rate, operation at voltages higher than 3.20 V would likely further decrease the HHV-based energy efficiency owing to additional activation and ohmic losses associated with higher cell voltages.

### 2.8. Economic Considerations of the Developed System

The total construction cost of the developed membrane-free flow-by alkaline electrolyzer was approximately R$1850.85 (USD 365.78). The electrolyte circulation system, consisting of two peristaltic pumps, was the largest cost component, accounting for R$1133.54 (USD 224.02), or 61.2% of the total. The monitoring and control system, comprising two Hall-effect flow sensors and an ESP32-S3 microcontroller, accounted for R$234.03 (USD 46.25), representing 12.6% of the total. The tubular electrolyzer, including the acrylic body, AISI 304 stainless-steel electrodes, additively manufactured PETG components, and the internal flow distributor, accounted for R$189.94 (USD 37.54), corresponding to 10.3% of the total investment. The flow-control assembly, comprising the check valve and two motorized actuators, contributed R$183.70 (USD 36.30), or 9.9%, whereas the tubing and hydraulic connections accounted for R$109.64 (USD 21.67), representing 5.9% of the total cost. Overall, the electrolyte circulation system accounted for more than half of the total construction cost, whereas the remaining components required for the proposed membrane-free flow-by electrolyzer represented 38.8% of the total investment. These results demonstrate that the electrolyzer can be constructed from commercially available materials at relatively low cost, supporting its application as a laboratory-scale reactor. 

## 3. Discussion

The polarization curves exhibited the characteristic electrochemical behavior of alkaline water electrolysis, with negligible current densities at low applied voltages followed by a progressive increase as the cell voltage increased. This response indicates a transition from an activation-controlled regime to one increasingly governed by charge transfer and ohmic phenomena. The measured cell voltage comprises the reversible potential and activation, ohmic, and concentration overpotentials, with activation and ohmic losses being the predominant contributions under typical alkaline operating conditions [32]. Likewise, the initial region of the polarization curve is primarily controlled by activation overpotentials, whereas ohmic losses become progressively more significant as current density increases [33].

The apparent electrical characterization provides quantitative support for interpreting the polarization curves. Increasing the KOH concentration from 0.50 to 2.00 mol L^−1^ increased the apparent electrical conductance from 1.515 to 3.285 S, reduced the apparent electrical resistance from 0.660 to 0.304 Ω, and increased the apparent electrical conductivity from 0.0653 to 0.1416 S cm^−1^. These results demonstrate that the higher current densities observed in the polarization curves were accompanied by substantially lower internal electrical losses and more efficient ionic transport throughout the electrolyzer. This interpretation is consistent with the findings of Niblett et al. [22], who showed that bubble accumulation increased the predicted cell voltage from approximately 2.9 to 3.3 V, with the increase predominantly associated with higher ohmic overpotentials rather than charge-transfer losses. Collectively, these findings indicate that minimizing internal electrical resistance while maintaining high electrolyte conductivity is essential for sustaining higher current densities and improving the electrochemical performance of membrane-free alkaline electrolyzers.

Although the maximum current density at 3.0 V (0.076 A cm^−2^) was lower than that reported for optimized membrane-free alkaline electrolyzers, the electrochemical trends observed in the present study are in good agreement with previous investigations. Manzotti et al. [18] reported representative membrane-free flow-by electrolyzers operating with concentrated KOH electrolytes (30 wt.%), nickel-based catalytic electrodes, and reduced interelectrode spacing, yielding substantially higher current densities than those obtained in the present work. Likewise, Dos Anjos, Reis, and Naveira-Cotta [33] demonstrated that the electrochemical response of membrane-free flow-by electrolyzers is governed by the combined effects of activation, ohmic, and mass-transfer overpotentials, with electrolyte conductivity playing a decisive role in current generation. Similarly, Malek et al. [34] observed that increasing electrolyte concentration enhances ionic conductivity, decreases cell resistance, and improves electrochemical performance. Consistent with these findings, increasing the KOH concentration from 0.50 to 2.00 mol L^−1^ in the present study progressively shifted the polarization curves toward higher current densities. Therefore, despite differences in absolute current densities resulting from distinct reactor configurations and operating conditions, the influence of electrolyte concentration on the polarization behavior observed in the present membrane-free tubular flow-by electrolyzer is fully consistent with the electrochemical trends reported in the literature.

The higher current densities observed at higher cell voltages indicate that the applied potential was sufficient to overcome the kinetic limitations of the hydrogen evolution reaction (HER) and oxygen evolution reaction (OER), resulting in enhanced electrochemical activity. This behavior has been attributed to the combined effects of activation, ohmic, and bubble-induced mass-transfer resistances during electrolysis [35]. Furthermore, the membrane-free configuration reduces internal ohmic resistance by eliminating the separator, although efficient hydrodynamic control remains essential to maintain stable operation and adequate gas separation [17]. Recent advances in membrane-free electrolyzer design further demonstrate that electrochemical performance depends not only on the intrinsic activity of the electrode materials but also on reactor architecture and electrolyte flow distribution. Whittingham et al. [36] developed a fully additively manufactured membrane-free electrolyzer and, using computational fluid dynamics (CFD), showed that optimized electrode geometry and flow-field design maximize electrode–electrolyte interaction while minimizing stagnant regions that impair bubble removal and mass transport. These findings reinforce that hydrodynamic design is an integral component of membrane-free electrolyzer optimization, complementing electrochemical operating parameters such as electrolyte concentration and applied voltage.

Increasing KOH concentration significantly improved the electrochemical performance of the membrane-free flow-by electrolyzer, as evidenced by higher current densities on the polarization curves and increased hydrogen production predicted by the response surface model. This behavior is primarily attributed to the higher ionic conductivity of KOH, which reduces electrolyte resistance and facilitates ionic transport, thereby improving overall cell performance [37,38]. This interpretation is supported by Makhanu, Nziu, and Maube [39], who experimentally demonstrated that increasing the KOH concentration from 10 to 30 wt.% progressively enhanced hydrogen production, with the average water mass loss at 5 V increasing from approximately 2.6% to 3.7%, owing to the improved electrical conductivity of the electrolyte. Likewise, Umair et al. [40] reported that increasing the KOH concentration from 30 to 33.3 wt.% increased alkaline electrolyzer efficiency from approximately 67.9% to 71.9%, further confirming the beneficial effect of enhanced ionic conductivity on electrochemical performance. Although these studies investigated different electrolyzer configurations, whereas the present work employed a membrane-free tubular flow-by electrolyzer, all consistently demonstrate that increasing electrolyte conductivity reduces ohmic limitations, facilitates ionic transport, and enhances hydrogen production under alkaline operating conditions.

Applied voltage was identified as the most influential operating parameter, in agreement with both the polarization curves and the ANOVA results. Higher operating voltages increased the electrochemical driving force for the HER and OER, resulting in greater current densities and hydrogen production rates. Similar electrochemical behavior has been reported in alkaline electrolyzers, where increasing operating voltage simultaneously intensifies activation, ohmic, and mass-transfer overpotentials as electrolysis proceeds [41,42].

The significant interaction between KOH concentration and applied voltage, identified by the ANOVA, indicates that the beneficial effect of increasing electrolyte conductivity becomes more pronounced under higher electrochemical driving forces. Similar interaction effects have been reported through response surface methodology, demonstrating that electrolyzer performance depends on the combined influence of operating variables rather than on their isolated effects [43,44]. Moreover, hydrogen production in membraneless alkaline electrolyzers has been shown to exhibit a nonlinear dependence on both current response and KOH concentration, reinforcing the coupled influence of electrolyte composition and applied voltage on hydrogen generation [45].

Electrolyte flow rate significantly influenced hydrogen production, confirming that hydrodynamic conditions are fundamental to the performance of membrane-free flow-by electrolyzers. The response surface analysis showed that hydrogen production decreased as the electrolyte flow rate increased across the investigated operating range, indicating that higher flow does not necessarily improve electrochemical performance. These observations align with the findings of Zoljalali et al. [46], who demonstrated that hydrodynamic optimization is a key factor governing the performance of membrane-free electrolyzers. Increasing the electrolyte flow rate from 30 to 80 mL h^−1^ promoted earlier bubble detachment, reduced bubble coalescence, and enabled stable electrolysis at higher current densities. Furthermore, optimizing the Tesla-valve geometry increased the hydrogen production frequency by approximately 13%, demonstrating that appropriate flow distribution can substantially enhance gas removal and mass transport without modifying the electrode material or electrolyte composition.

These observations can be explained by the fundamental role of electrolyte circulation in membrane-free electrolyzers. Electrolyte flow simultaneously promotes ionic transport, gas separation, and bubble removal while preventing excessive gas accumulation on electrode surfaces, thereby supporting stable electrochemical operation. In membrane-free systems, efficient hydrodynamic control is particularly important because gas separation must occur without a physical membrane [33,47]. Hashemi et al. [23] demonstrated that electrolyte flow governs convection, ionic migration, bubble removal, and inertial gas separation, whereas O’Neil et al. [48] showed that increasing the fluid velocity from 0 to 6.6 cm s^−1^ substantially increased the electrolysis current and eliminated current oscillations associated with bubble accumulation. However, further increasing the velocity to 13.2 cm s^−1^ produced only marginal performance improvements, indicating the existence of an optimal hydrodynamic condition beyond which additional increases in flow provide limited electrochemical benefits.

The lower hydrogen production observed at higher electrolyte flow rates suggests that the benefits of enhanced bubble removal were offset by the reduced electrolyte residence time within the electroactive region. Increasing flow velocity improves bubble removal and reduces mass-transfer overpotentials; however, its electrochemical benefits gradually diminish beyond an optimum operating condition [41,42,49]. In addition, the performance of membrane-free electrolyzers strongly depends on flow distribution, electrode arrangement, and gas-separation design, while the interaction between gas–liquid two-phase flow and electrochemical reactions governs current-density distribution and overall reactor performance [50,51]. These mechanisms help explain why the lowest investigated electrolyte flow rate provided the highest hydrogen production, indicating that the tubular flow-by configuration achieved a more favorable balance between ionic transport, bubble removal, and electrolyte residence time.

The ANOVA confirmed that KOH concentration, applied voltage, and electrolyte flow rate significantly affected hydrogen production, while the Pareto chart identified applied voltage as the dominant operating variable, followed by electrolyte concentration and electrolyte flow rate. The significance of the interaction and quadratic terms further indicates that hydrogen production is governed by nonlinear electrochemical and hydrodynamic phenomena rather than by simple linear relationships.

The combined use of ANOVA and response surface methodology has proven effective for identifying the relative importance of operating variables and their interactions in alkaline water electrolysis, enabling the determination of optimal operating regions and the interpretation of nonlinear system behavior [43]. Likewise, the coupled influence of electrochemical and hydrodynamic parameters on current density and specific energy consumption underscores the importance of optimizing multiple operating variables simultaneously rather than evaluating them independently [44]. A comparable statistical strategy was reported by Majasan et al. [52], who applied a full-factorial design-of-experiments combined with ANOVA and regression analysis to investigate mass-transport parameters in a polymer electrolyte membrane electrolyzer. The authors demonstrated that the experimental design successfully identified both individual effects and interactions among operating variables, yielding a predictive model with excellent goodness-of-fit (R^2^ = 99.64%). These findings reinforce the suitability of factorial experimental design as a robust statistical tool for evaluating complex electrochemical systems governed by multiple interacting operating parameters. This conclusion is further supported by Makhanu, Nziu, and Maube [39], who employed a full factorial experimental design comprising 48 experimental runs combined with response surface methodology to optimize alkaline water electrolysis. Their study demonstrated that simultaneous evaluation of applied voltage, electrolyte concentration, and electrode spacing provides a more comprehensive understanding of hydrogen production than analyses based on isolated operating variables. Similarly, the present work confirms that the interaction among KOH concentration, electrolyte flow rate, and applied voltage governs hydrogen production in membrane-free tubular electrolyzers, highlighting the robustness of factorial experimental design for electrochemical process optimization.

The response surfaces obtained in the present study clearly reflect these coupled effects, demonstrating that hydrogen production increased with increasing KOH concentration and applied voltage, whereas increasing electrolyte flow shifted the response toward lower hydrogen production within the investigated experimental domain. Moreover, the high coefficients of determination together with the close agreement between predicted and experimental values confirmed the robustness and predictive capability of the quadratic model. Similar conclusions have been reported for hydrogen production systems, where quadratic response surface models incorporating linear, interaction, and quadratic effects provide reliable prediction and optimization of process performance [53,54].

The optimized operating condition resulted from the combined effects of increased electrolyte conductivity from the higher KOH concentration, favorable hydrodynamic conditions, and the higher applied voltage, as evidenced by the high desirability value and the close agreement between the predicted and experimentally validated hydrogen production rates. These results indicate that the quadratic response surface model successfully identified the optimal operating region within the investigated experimental domain. From a mechanistic perspective, this optimum reflects the balance among enhanced ionic transport promoted by the higher KOH concentration, the greater electrochemical driving force imposed by the applied voltage, and an electrolyte flow rate sufficient to facilitate bubble removal while preserving effective electrolyte–electrode interaction. Together, these factors reduce ohmic resistance and improve mass transport, thereby maximizing hydrogen production under the investigated operating conditions. Simultaneous optimization of operating variables has been shown to maximize hydrogen production while minimizing energy consumption, highlighting the importance of evaluating the combined influence of electrochemical and operating parameters rather than optimizing each variable individually [41]. These findings are also consistent with previous reports demonstrating that hydrogen production in membraneless alkaline electrolyzers is governed by the coupled influence of electrolyte concentration and electrochemical response, reinforcing the optimized operating condition identified in the present study [55].

The electrochemical indicators obtained under the optimal operating conditions provide a more comprehensive assessment of the proposed electrolyzer’s performance. The apparent Faradaic efficiency of approximately 88.7% indicates that most of the electrical charge supplied to the system was effectively converted into hydrogen, despite the absence of a physical membrane and the associated challenges of gas crossover, bubble accumulation, and two-phase transport. However, the specific energy consumption of 8.63 kWh Nm^−3^ H_2_, corresponding to energy efficiencies of approximately 41.0% based on the HHV and 34.1% based on the LHV, indicates that a substantial fraction of the supplied energy was still dissipated through activation, ohmic, and mass-transfer losses. Therefore, although the Faradaic efficiency confirms satisfactory charge utilization and reliable hydrogen generation, the energetic indicators reveal that further improvements in electrode geometry, interelectrode spacing, electrical resistance, and hydrodynamic control are required to increase the overall energy efficiency. These results are consistent with the performance expected for a low-cost, bench-scale, membrane-free electrolyzer and demonstrate that the proposed system is technically suitable for hydrogen production while also identifying the main opportunities for future optimization [35,37,56,57].

## 4. Materials and Methods

### 4.1. Experimental Strategy

The present study was developed in four main stages: (i) design and construction of a laboratory-scale tubular alkaline electrolyzer operating in a membrane-free flow-by configuration; (ii) development of a gas–liquid separation system and volumetric measurement of the produced gases; (iii) electrochemical characterization of the system by means of polarization curves; and (iv) evaluation of the effects of the operating conditions on hydrogen production through experimental design and statistical analysis.

The adopted approach enabled the simultaneous evaluation of the constructive feasibility of the system, its electrochemical performance, the hydrodynamic behavior of the flow and the hydrogen production efficiency.

### 4.2. Construction of the Tubular Alkaline Electrolyzer

A laboratory-scale tubular alkaline electrolyzer operating in a membrane-less flow-by configuration, without a membrane or internal diaphragm, was developed. In this architecture, the electrolyte continuously circulates through the electroactive region, promoting the transport of the gaseous products generated during electrolysis to the external gas–liquid separation system. Systems of this nature have been investigated as lower-complexity alternatives that could reduce costs associated with conventional membranes and separators [34]. The electrolytic cell was constructed from a transparent acrylic tube with an internal length of approximately 25 cm and an internal diameter of 50 mm, while the end caps, supports, fittings, and sealing elements were manufactured via additive manufacturing (3D printing).

The electrolyte was introduced through the lower section of the reactor, promoting upward flow through the electroactive region. To ensure unidirectional electrolyte flow and prevent backflow caused by hydraulic oscillations in the system, a check valve was installed in the lower feed line.

**Figure 10:**
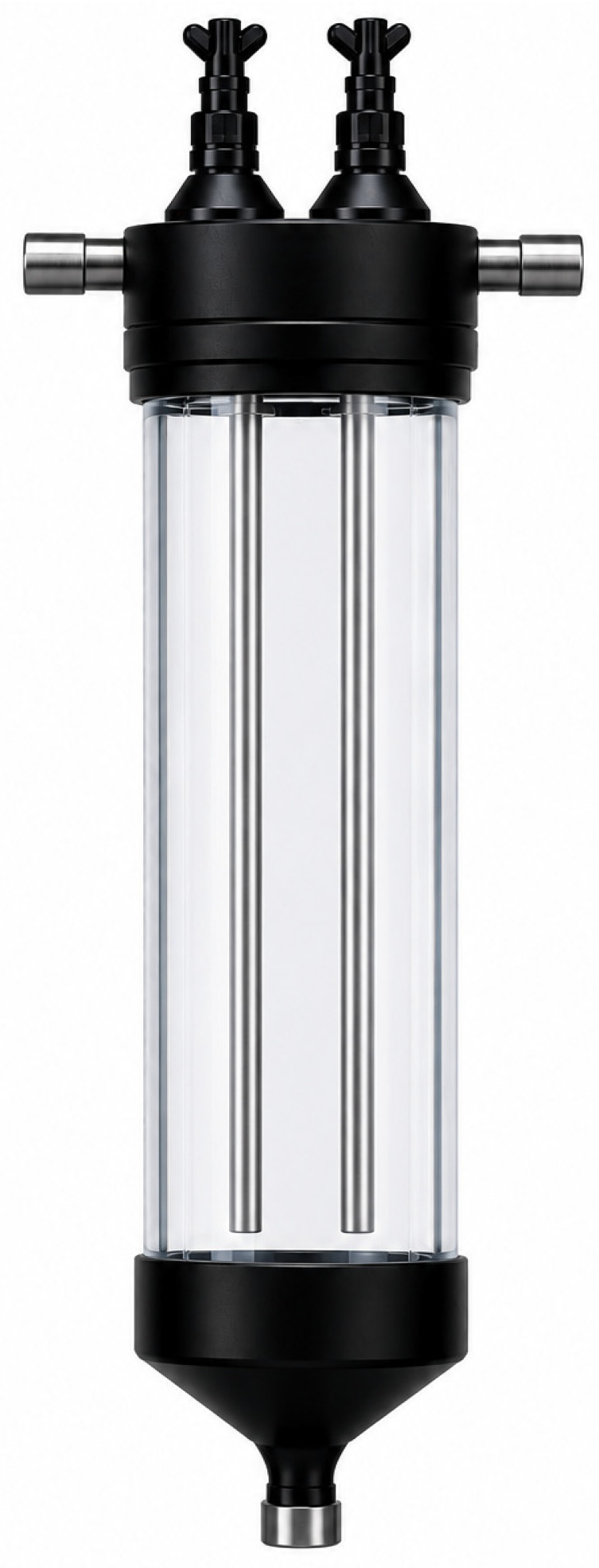
Schematic configuration of the membrane-less flow-by tubular alkaline electrolyzer used in the experiments.

At the upper end of the cell, two independent outlets were installed to continuously remove the two-phase flows originating from the anodic and cathodic regions. This configuration was adopted to promote bubble detachment during electrolysis, minimize gas retention inside the cell, and improve the hydrodynamic operating conditions. The structural configuration of the tubular alkaline electrolyzer developed in this study is presented in Figure 10.

The electrodes were fabricated from AISI 304 stainless steel as cylindrical rods, with a total length of 26.5 cm and a diameter of 0.6 cm. The selection of this material was motivated by its high chemical and mechanical stability in alkaline media, good electrical conductivity, wide commercial availability, and low cost compared with conventional electrocatalytic materials. Additionally, the presence of elements such as iron, nickel, and chromium in its composition has been associated with promising electrocatalytic behavior in alkaline water electrolysis, making stainless steel an attractive alternative for developing low-cost electrolyzers [58]. During operation, approximately 22 cm of each electrode remained immersed in the electrolyte, constituting the electroactive region of the system. The operational electrolyte volume used during the experiments was 2.5 L. The main geometric and construction specifications of the tubular alkaline electrolyzer developed in this study are summarized in Table 4.

### 4.3. Gas–Liquid Separation System and Gas Measurement

A low-cost system was developed for gas–liquid separation and volumetric quantification of the gases produced during electrolysis. The device was manufactured via 3D printing and designed with two independent inlets connected to the electrolyzer’s upper outlets. One inlet received the two-phase flow from the cathodic region, consisting of electrolyte and hydrogen, while the other received the two-phase flow from the anodic region, consisting of electrolyte and oxygen.

**Figure 11:**
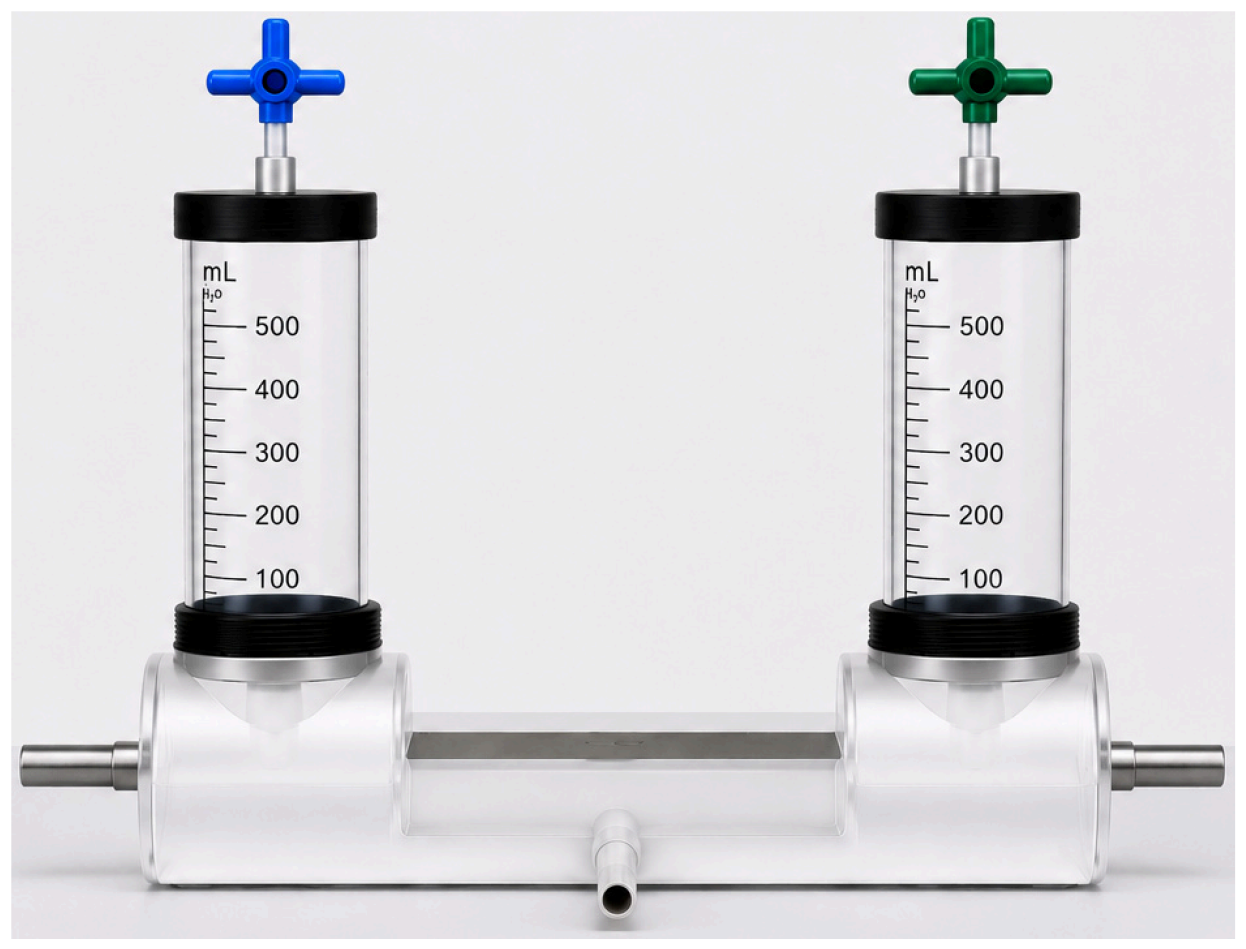
Schematic configuration of the gas–liquid separation and volumetric gas measurement system.

Internally, the device was divided into two independent compartments, each containing separation chambers equipped with internal baffles to increase the residence time of the gas–liquid mixture and promote gravitational phase separation. The structural configuration of the gas–liquid separation and volumetric gas measurement system developed in this study is presented in Figure 11.

After separation, the electrolyte was directed to a common bottom outlet connected to the main reservoir, allowing its continuous recirculation. The separated gases were conveyed to individual collectors fabricated from acrylic tubes with an internal diameter of 50 mm, in which gas production was determined based on the liquid–gas displacement principle. In this method, the produced gas displaced an equivalent volume of electrolyte previously contained within the collector, allowing the direct determination of the gas volume produced throughout the experimental run. Volumetric displacement-based methods are widely employed for the experimental quantification of hydrogen production in electrolysis systems due to their operational simplicity and high reliability for laboratory-scale measurements [59]. Pressure relief valves were installed at the top of the collectors to allow the controlled release of the accumulated gases at the end of each experimental run, preventing pressure buildup within the system.

### 4.4. Instrumentation and Data Acquisition

The electrolyte circulation was provided by a peristaltic pump with an operating flow range of 0–6 L min^−1^. The electrolyte flow rates in the anodic and cathodic circulation lines were continuously monitored using two turbine-type Hall-effect flow sensors (YF-B1-S), each installed in an independent flow line. The flow sensors generated pulse signals proportional to the instantaneous volumetric flow rate, which were processed by an ESP32-S3 microcontroller equipped with an integrated TFT display for real-time data acquisition and visualization.

Unlike conventional monitoring systems, the flow measurement unit operated under a closed-loop automatic control strategy. Two motorized stainless-steel ball valves were installed in the anodic and cathodic outlet lines to independently regulate the electrolyte flow delivered to each side of the electrolyzer. Based on the real-time signals from the flow sensors, the ESP32-S3 continuously compared the measured flow rates with the predefined setpoints and actuated the motorized valves via a four-channel 5 V relay module. This configuration enabled automatic balancing and stabilization of the electrolyte flow in both circulation lines throughout the experimental operation, minimizing hydraulic fluctuations and improving the repeatability of the electrolysis tests.

Electrical power was supplied by a programmable DC power supply (0–15 V, 0–80 A). The electrical parameters of the electrolyzer were continuously monitored using a digital wattmeter installed between the programmable DC power supply and the electrolyzer, allowing real-time measurement and recording of the applied voltage, electric current, and power consumption during each experimental run.

An approximate cost analysis was conducted to estimate the contribution of the main components to the total construction cost of the developed system. The analysis considered only components directly associated with the construction and operation of the proposed electrolyzer, including the reactor, electrolyte circulation, flow control, and monitoring systems. General-purpose laboratory equipment, such as bench power supplies used to energize the electrolyzer and peristaltic pumps, was excluded because these devices constitute auxiliary laboratory infrastructure rather than intrinsic components of the proposed design. The contribution of each component was determined by the ratio of its approximate acquisition cost to the system’s total construction cost. Component costs were estimated from the acquisition prices of commercially available materials and devices at the time the experimental setup was assembled. Currency conversion was performed using an exchange rate of USD 1 = R$5.06.

**Figure 12:**
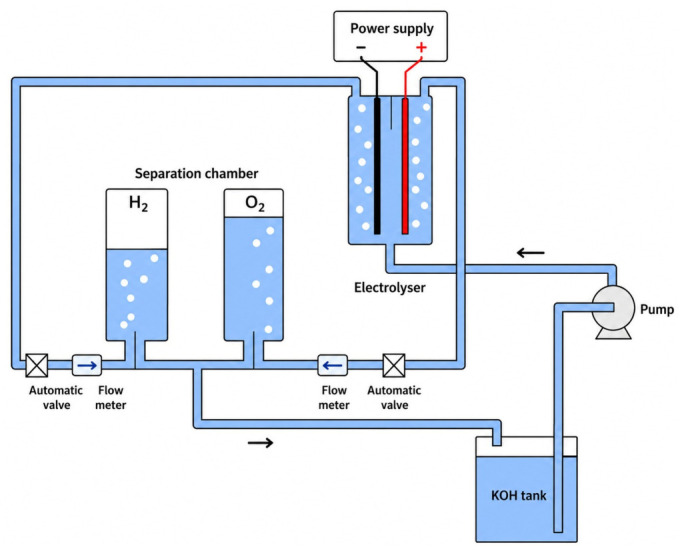
Schematic representation of the complete continuous-flow alkaline water electrolysis system, including the electrolyzer, gas–liquid separator, electrolyte recirculation circuit, flow-control unit, and gas collection system.

The complete experimental setup, including the electrolyte recirculation circuit, the membrane-less flow-by electrolyzer, the gas–liquid separation system, the automatic flow-control unit, and the volumetric gas collectors, is schematically illustrated in Figure 12.

### 4.5. Hydraulic and Operational Validation

Before the experimental tests, the system was subjected to a hydraulic and operational validation stage. Initially, the assembly was operated without applying electrical voltage to evaluate leak tightness of the connections, the absence of leaks, the operation of the peristaltic pump, flow stability, the response of the flow sensors, and the operation of the data acquisition system. Subsequently, tests were performed with the application of electrical voltage to verify bubble formation at the electrodes, the transport of the two-phase flows, the efficiency of the gas–liquid separation process, the response of the gas collectors, and the repeatability of the volumetric measurements. The system was considered validated when it exhibited stable operation, no leaks, and satisfactory measurement repeatability.

### 4.6. Electrolyte Preparation

Aqueous potassium hydroxide (KOH) solutions were prepared by dissolving the reagent in distilled water. KOH was selected as the electrolyte for its high ionic conductivity, widespread use in alkaline electrolysis systems, and ability to promote hydroxide-ion transport in the reaction medium. Additionally, electrolyte concentration is a key operational parameter that affects electrolysis performance. Increasing the KOH concentration enhances the solution’s electrical conductivity, thereby promoting higher current densities and hydrogen production rates [60]. Solutions were prepared by gradually adding KOH under continuous stirring until complete dissolution. After preparation, solutions were allowed to reach room temperature (25 ± 2 °C) before use. All experiments were conducted without external heating to preserve simple, reproducible laboratory conditions consistent with the low-cost design of the proposed system. Although higher operating temperatures may enhance electrolyte conductivity and electrode kinetics, their use would require additional thermal control and auxiliary energy consumption.

### 4.7. Electrochemical Characterization by Polarization Curves

The electrochemical characterization of the electrolyzer was performed by obtaining polarization curves, which are widely used to evaluate the performance of alkaline electrolysis systems. Polarization curves show the relationship between applied voltage and resulting current density, providing insight into the cell’s electrochemical behavior under different operating conditions. Furthermore, they constitute an important tool for comparing system performance and defining the operating conditions employed in the experimental tests [61]. The experiments were conducted in a flow-by configuration, with the electrolyte flow rate maintained at 2.50 L min^−1^. Before each test, the electrolyte was circulated for approximately 5 min to ensure hydraulic stabilization and system homogenization. Subsequently, the applied voltage was gradually increased from 1.6 to 3.0 V, allowing 60 to 120 s for current stabilization at each experimental point. During the experiments, the applied voltage and electric current values were recorded.

### 4.8. Apparent Electrical Characterization

To complement the electrochemical evaluation provided by the polarization curves, an apparent electrical characterization of the electrolyzer was performed using the approximately linear region of the current–voltage relationship. This complementary analysis was adopted to provide quantitative electrical parameters that facilitate comparison of the overall electrical behavior of the electrolyzer under different electrolyte concentrations. The proposed approach enables characterization of variations in the apparent electrical response of the cell directly from experimentally measured polarization data, providing additional indicators for interpreting the influence of electrolyte composition on the electrical performance of the system. 

The apparent electrical characterization was performed using the average current values obtained from three independent polarization experiments for each experimental condition. The current–voltage data corresponding to the approximately linear region of the polarization curves were selected and fitted by linear regression using Equation (4), allowing determination of the apparent electrical conductance from the slope of the fitted line.

(4) I=GV+b

where I is the measured current (A), V is the applied cell voltage (V), G is the apparent electrical conductance (S), defined in this work as the slope of the linear regression, and b is the intercept. The apparent cell resistance $R_{app}$ was subsequently calculated as the reciprocal of the apparent electrical conductance (Equation (5)):

(5) $$R_{app}=\frac{1}{G}$$

Following the classical relationship between electrical conductance and resistance. Likewise, an apparent geometric conductivity $\kappa_{app}$ was estimated from the apparent conductance and the geometric cell constant according to Equation (6):

(6) $$\kappa_{app}=G\left(\frac{d}{A}\right)$$

where *d* is the interelectrode distance (1.80 cm), *A* is the effective electrode area (41.75 cm^2^), and $\frac{d}{A}$ corresponds to the geometric cell constant. The resulting cell constant for the present electrolyzer was 0.0431 cm^−1^. These relationships are consistent with the classical theory of conductometric measurements, in which electrical conductivity is obtained from the measured conductance and the geometry of the conductivity cell [62].

Because this procedure is derived from the global current–voltage response of the electrolyzer, the calculated parameters represent apparent electrical properties of the system. These indicators provide a complementary quantitative description of the electrolyzer’s electrical characteristics, enabling direct comparison of the effects of electrolyte concentration on the apparent conductance, resistance, and geometric conductivity measured under identical experimental conditions. Such parameters serve as practical descriptors of the overall electrical behavior of the developed electrolyzer and complement the conventional interpretation of polarization curves [63]. 

### 4.9. Experimental Design

The operational optimization of the system was carried out using a full factorial 3^3^ experimental design supplemented with six replicates at the center point. This approach was adopted because it allows the simultaneous evaluation of the main effects and interactions among the investigated operating variables, overcoming the limitations associated with the one-factor-at-a-time approach. Factorial experimental designs have been widely employed for process investigation and optimization, enabling the identification of the most influential factors affecting the system response and their respective interactions [52,64]. The investigated factors and their corresponding coded and actual levels are presented in Table 5.

A total of 27 factorial experiments and six replicates at the center point were performed, resulting in 33 experimental runs. The response variable adopted was the hydrogen production rate. The experimental matrix corresponding to the full factorial 3^3^ design, including the coded levels and the corresponding actual values of the investigated variables, is presented in Table 6.

The six replicates performed at the center point were used to estimate the pure experimental error, evaluate the reproducibility of the system, and verify the presence of curvature in the response surface. The experimental run order was randomized before conducting the experiments to minimize possible systematic errors associated with electrode conditioning, electrolyte heating, and operational variations over time.

### 4.10. Experimental Procedure

For each experimental run, the corresponding KOH solution was added to the system reservoir. The peristaltic pump flow rate was set to the established experimental condition, and the ball valves in the anodic and cathodic lines were adjusted. After hydraulic stabilization for approximately 3–5 min, the corresponding test voltage was applied. After an operational stabilization period of approximately 60 s, hydrogen production was quantified by volumetric displacement. During each experimental run, the displaced gas volume, collection time, applied voltage, electric current, electrical power consumption, and operational observations were recorded. At the end of each experiment, the accumulated gases were released through the pressure relief valves, and the system was prepared for the subsequent experimental condition.

To complement the electrochemical characterization, additional electrochemical and energy-performance indicators were calculated from the experimentally measured voltage, current, and hydrogen production data. The calculations were based on Faraday’s law, the ideal gas law, and conventional electrolyzer performance definitions reported in the literature [33,40,56,65,66].

The hydrogen production rate was calculated from the ratio between the collected gas volume and the corresponding collection time, according to Equation (7):

(7) $${\dot{V}}_{H_{2}}\;=\frac{∆V}{∆t}$$

where ∆V is the measured hydrogen volume and ∆t is the corresponding collection time.

The current density was determined from the ratio between the measured current and the immersed geometric area of the cylindrical cathode, according to Equation (8):

(8) $$j=\frac{I}{A_{geo}}$$

where I is the measured current and $A_{geo}$ is the immersed geometric area of the cathode.

Faradaic efficiency was calculated according to Equation (9):

(9) $$\eta_{F\;}=\frac{{2F\dot{n}}_{H_{2}}}{I}\times 100$$

where F is Faraday’s constant (96.485 C mol^−1^) and ${\dot{n}}_{H_{2}}$ is the hydrogen molar production rate obtained from the experimentally measured gas flow using the ideal gas law.

The cell-specific electrical energy consumption was calculated according to Equation (10):

(10) $${SEC}_{cell}=\frac{VI}{1000\;{\dot{V}}_{H_{2},N}}$$

where V is the applied cell voltage, I is the electric current, and ${\dot{V}}_{H_{2},N}$ is the hydrogen production rate normalized to standard conditions (Nm^3^ h^−1^). The resulting ${SEC}_{cell}$ is expressed in kWh Nm^−3^ H_2_. The HHV-based energy efficiency was calculated as (Equation (11):

(11) $$\eta_{HHV\;}=\frac{{\dot{n}}_{H_{2}}{HHV}_{H_{2}}}{VI}\;\times \;100$$

where ${HHV}_{H_{2}}$ is the higher heating value of hydrogen and VI is the electrical power supplied to the electrolyzer.

### 4.11. Statistical Data Analysis

The experimental data were processed using Python 3.14.6 [67]. A quadratic polynomial regression model was fitted by least squares within the framework of Response Surface Methodology (RSM) to describe how KOH concentration, electrolyte flow rate, and applied voltage affect the hydrogen production rate. The statistical significance of the model terms was evaluated by analysis of variance (ANOVA), including a curvature test, at the 95% confidence level (*p* < 0.05). The relative importance of the factors and their interactions was further assessed using standardized Pareto charts. The adequacy of the fitted model was evaluated using the coefficients of determination (R^2^, adjusted R^2^, and predicted R^2^), the root mean square error (RMSE), the observed-versus-predicted values plot, and residual analysis. The validated model was then used to generate response surfaces and contour plots and to perform numerical optimization using the desirability function to determine the operating conditions that maximize the hydrogen production rate. Finally, the electrochemical and energy-performance indicators described in Section 4.9 were calculated for the selected operating condition to provide experimental validation and performance characterization.

## 5. Conclusions

This work developed, fabricated, and experimentally validated a low-cost membrane-less tubular flow-by alkaline electrolyzer integrated with a dedicated gas–liquid separation and volumetric gas-measurement system. The proposed platform enabled reliable electrochemical characterization through polarization curves and provided a comprehensive framework for evaluating the combined effects of electrolyte concentration, electrolyte flow rate, and applied voltage on hydrogen production using statistical experimental design and response surface methodology.

The integration of reactor development, electrochemical characterization, apparent electrical analysis, and statistical optimization demonstrated that the proposed methodology is suitable for systematically investigating the performance of membraneless alkaline electrolyzers. The developed quadratic model showed satisfactory predictive capability and proved to be an effective tool for interpreting the influence of the investigated operating variables and for defining operating conditions within the studied experimental domain.

The operating condition corresponding to 2.00 mol L^−1^ KOH, 1.00 L min^−1^ electrolyte flow rate, and 3.20 V should be interpreted exclusively as the optimum identified for the developed prototype within the experimental region investigated. This result should not be extrapolated to a generally optimal operating voltage for conventional alkaline water electrolysis systems, which typically operate at lower cell voltages and under different design, materials, and operating conditions. Rather, it defines the operating point at which the proposed membrane-less reactor achieved its highest hydrogen production under the selected experimental conditions.

Overall, the main contribution of this study is the establishment of an experimentally validated, low-cost, and modular platform that integrates reactor development, gas management, electrochemical characterization, and statistical optimization into a single methodological framework. This approach provides a reproducible basis for future investigations aimed at improving membrane-less alkaline electrolysis through advances in electrode materials, reactor geometry, hydrodynamic design, operating strategies, and scale-up, thereby contributing to the development of more efficient and economically accessible technologies for sustainable hydrogen production.

## Figures and Tables

**Table 1:** Apparent electrical parameters obtained from linear regression of the polarization curves.

KOH (mol L^−1^)	Apparent Conductance, G (S)	Apparent Resistance, $R_{app}$ (Ω)	Apparent Conductivity, $\kappa_{app}$ (S cm^−1^)	R^2^
0.50	1.515	0.660	0.0653	0.9992
1.25	2.485	0.402	0.1071	0.9965
2.00	3.285	0.304	0.1416	0.9967

**Table 2:** Analysis of variance (ANOVA) for the quadratic response surface model.

Source	df	SS	MS	*F*-Value	*p*-Value
Model	9	0.407316	0.045257	63.72	<0.0001
KOH concentration (A)	1	0.070576	0.070576	99.36	<0.0001
Electrolyte flow rate (B)	1	0.024614	0.024614	34.65	<0.0001
Applied voltage (C)	1	0.273124	0.273124	384.53	<0.0001
A × B	1	0.002622	0.002622	3.69	0.0672
A × C	1	0.022204	0.022204	31.26	<0.0001
B × C	1	0.005375	0.005375	7.57	0.0114
A^2^	1	0.002285	0.002285	3.22	0.0861
B^2^	1	0.002832	0.002832	3.99	0.0578
C^2^	1	0.003873	0.003873	5.45	0.0286
Residual	23	0.016337	0.000710	—	—
Lack-of-Fit	17	0.015817	0.000930	10.74	0.0038
Pure Error	6	0.000520	0.0000866	—	—
Total	32	0.423652	—	—	—

**Table 3:** Experimental electrochemical and energy-performance indicators obtained at the best operating condition identified within the investigated domain.

Parameter	Unit	Value
KOH concentration	mol L^−1^	2.00
Electrolyte flow rate	L min^−1^	1.00
Applied voltage	V	3.20
Hydrogen production rate	mL s^−1^	0.4395
Current density	A cm^−2^	0.0943
Faradaic efficiency	%	88.7
Cell-specific electrical energy consumption	kWh Nm^−3^ H_2_	8.63
Energy efficiency, HHV basis	%	41.0

**Table 4:** Construction characteristics of the tubular alkaline electrolyzer.

Parameter	Specification
Operating configuration	Flow-by
Cell type	Membrane-less alkaline
Cell body material	Acrylic
Internal length	25 cm
Internal diameter	50 mm
Electrolyte inlet	Bottom
Check valve	Yes
Upper outlets	2
Electrode material	AISI 304 stainless steel
Electrode geometry	Cylindrical rods
Total electrode length	26.5 cm
Immersed length	22 cm
Electrode diameter	0.6 cm

**Table 5:** Experimental matrix of the investigated factors and their corresponding coded and actual levels.

Factor	−1	0	+1
KOH Concentration (mol L^−1^)	0.50	1.25	2.00
Electrolyte Flow Rate (L min^−1^)	1.00	2.50	4.00
Applied Voltage (V)	2.20	2.70	3.20

**Table 6:** Experimental matrix of the full factorial 3^3^ design with the coded and actual levels of the investigated variables.

Run	KOH Concentration (Cod.) *	Electrolyte Flow Rate (Cod.)	Applied Voltage (Cod.)	KOH Concentration (mol L^−1^)	Electrolyte Flow Rate (L min^−1^)	Applied Voltage (V)
1	−1	−1	−1	0.50	1.00	2.20
2	−1	−1	0	0.50	1.00	2.70
3	−1	−1	+1	0.50	1.00	3.20
4	−1	0	−1	0.50	2.50	2.20
5	−1	0	0	0.50	2.50	2.70
6	−1	0	+1	0.50	2.50	3.20
7	−1	+1	−1	0.50	4.00	2.20
8	−1	+1	0	0.50	4.00	2.70
9	−1	+1	+1	0.50	4.00	3.20
10	0	−1	−1	1.25	1.00	2.20
11	0	−1	0	1.25	1.00	2.70
12	0	−1	+1	1.25	1.00	3.20
13	0	0	−1	1.25	2.50	2.20
14	0	0	0	1.25	2.50	2.70
15	0	0	+1	1.25	2.50	3.20
16	0	+1	−1	1.25	4.00	2.20
17	0	+1	0	1.25	4.00	2.70
18	0	+1	+1	1.25	4.00	3.20
19	+1	−1	−1	2.00	1.00	2.20
20	+1	−1	0	2.00	1.00	2.70
21	+1	−1	+1	2.00	1.00	3.20
22	+1	0	−1	2.00	2.50	2.20
23	+1	0	0	2.00	2.50	2.70
24	+1	0	+1	2.00	2.50	3.20
25	+1	+1	−1	2.00	4.00	2.20
26	+1	+1	0	2.00	4.00	2.70
27	+1	+1	+1	2.00	4.00	3.20
28	0	0	0	1.25	2.50	2.70
29	0	0	0	1.25	2.50	2.70
30	0	0	0	1.25	2.50	2.70
31	0	0	0	1.25	2.50	2.70
32	0	0	0	1.25	2.50	2.70
33	0	0	0	1.25	2.50	2.70

* Coded levels correspond to −1 (low level), 0 (center point), and +1 (high level) for KOH concentration, electrolyte flow rate, and applied voltage.

## Data Availability

The original contributions presented in the study are included in the article; further inquiries can be directed to the corresponding author.
